# Minimally invasive spine surgery: evaluation of clinical and functional outcomes and their correlation with the return to work

**DOI:** 10.47626/1679-4435-2020-518

**Published:** 2020-12-11

**Authors:** Anibal Correia Silva, Tabata Alcantara

**Affiliations:** 1 Ortopedia, Universidade Potiguar - Natal (RN), Brazil

**Keywords:** spine, minor surgical procedures, occupational medicine, return to work

## Abstract

**Background:**

Intervertebral disc changes are a multi-factorial problem whose main clinical feature is pain. Studies show that when clinical treatments fail, the proposed surgical treatments frequently present unsatisfactory results. Traditional lumbar arthrodesis causes important clinical and functional changes that can result in complications and jeopardize the patients’ quality of life.

**Objectives:**

This study aims to investigate the clinical and functional results of minimally invasive spine surgery in patients with a clinical diagnosis of low-back or sciatic pain and segmental instability, finally correlating these results with the patients’ return to work.

**Methods:**

Patients signed an informed consent form and were clinical and radiographically re-evaluated by independent professionals in the pre- and postoperative periods. Evaluation methods used the Oswestry disability index, as well as visual analog scale and Medical Outcomes Short Form Health Survey (SF-36) scores. We also retrieved epidemiological data, information on work resumption, and bone consolidation evaluations from the medical records.

**Results:**

We evaluated 19 patients who had been operated on 33 levels; visual analog scale and Oswestry disability index scores were initially reduced from 10% to 2% and from 64% to 28%, respectively. SF-36 scores were significantly higher in 5 of the 7 questionnaire scales at the end of the follow-up period. Most patients (68.4%) did not return to work after surgery; the others returned 2 to 67 months after the procedure. All patients received social security benefits after the surgery.

**Conclusion:**

Although the procedure presented positive results, it did not result in a satisfactory return-to-work rate. Our results should be analyzed in view of the low educational level and income of the patients, the manual nature of their labor, and the validity of social security benefits.

## INTRODUCTION

Low-back pain is an important cause of work incapacity in the Western world; it reaches 80% to 90% of the population and is responsible for an alarming rate of absenteeism and loss of workforce resources within the economically active population. Its resolution is difficult and clinical treatments often present unsatisfactory results.^1^^,^^2^ Studies that investigated surgical treatments have also presented limited results, especially considering patients that were eligible for social security benefits: These patients had twice the chances of achieving unsatisfactory results after surgery when compared to those that did not receive these benefits.^3^

The introduction of video-assisted surgery for resection of the intervertebral disc represented an important achievement.^4^ Although lumbar arthrodesis is an established procedure used in the treatment of various vertebral lesions,^5^ being considered the gold standard among invasive treatments for low-back pain, it has been shown to be an aggressive procedure that leads to blood loss and muscular injury, in addition to resections of laminae, ligamenta flava, and facet joints.^6^

Considering the complications that derive from this classic technique, could minimally invasive spine surgery benefit workers in their return to work? This study investigates whether this procedure provides the same results as conventional lumbar arthrodesis, that is, spinal fusion with good clinical and functional results that allow workers to return to work promptly.

## METHODS

Our inclusion criteria were chronic low-back or sciatic pain associated with segmental instability, according to Panjabi.^7^ Our patients had not responded to effective clinical treatment for at least 6 weeks,^8^ presenting the classical indications for conventional arthrodesis; they were instead subjected to minimally invasive spine surgery.

All patients were cared for at OrtoClin ambulatory clinic, in Natal, state of Rio Grande do Norte, by the author between April 2009 and July 2015. We excluded patients with tumor diagnoses, infections, and those who had had previous surgeries or minimally invasive spine surgery but required different reinterventions.

Patients were clinical and radiographically evaluated by independent professionals in the pre- and postoperative periods through visual analog scale (VAS)^9^ scores, the Oswestry disability index (ODI),^10^ and Medical Outcomes Short Form Health Survey (SF-36) scores.^11^ Surgical (operative time, length of stay, need for blood transfusion) and demographic data, as well as information regarding work resumption, were retrieved from the patients’ medical records. Vertebral arthrodesis was confirmed through computed tomography imaging showing trabecular bone between vertebrae.^12^ All patients were informed of the study objectives and signed a free and informed consent form approved by the Ethics Committee of the Institute for the Medical Assistance of State Government Employees (IAMSPE).

### STATISTICAL ANALYSIS

The collected data were submitted to statistical analysis expressing categorical variables in absolute (n) and relative (%) values, and continuous variables were verified through a Shapiro-Wilk test. Variables that presented normal distributions were expressed using mean (SD) and compared using a Student’s t-test. Variables that did not have this distribution were expressed as median and quartiles (Q25-Q75), and were analyzed with a Wilcoxon signed-rank test. Pain perception at three different moments (before surgery, 1 week after surgery and 3 years after surgery) was compared using the Friedman test, and the binary correlation between continuous variables was measured using the Spearman’s product-moment test. Statistical significance considered p < 0.05 for all analyses, which were performed using the SPSS statistical package v. 25.0.

## RESULTS

**Figure 1:**
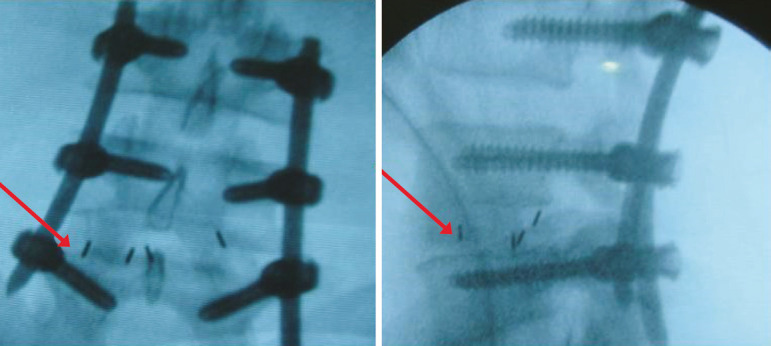
X-ray images showing percutaneous screws and vertebral prostheses (indicated by arrows).

Starting from an initial sample size of 24, we lost 5 patients (4 requiring reinterventions in other services and one death due to urban violence); among the remaining 19 patients, 17 were male and 2 were female, with mean ages of 36.1 (SD, 1.2) years old. The mean follow-up period was 47 (range, 29-70) months, with clinical progression prior to surgery lasting 40.3 (7-132) months. The mean period of effective clinical treatment (when the patient was under our clinical care) before surgery was 18.4 (3-72) months. We operated on 33 spinal segments, all comprised between L2 and S1; 73.7% of the cases involved 2 segments. We used 8-and 10-mm vertebral prostheses^13^ according to pre-introduction fittings and in accordance with the patient’s physical constitution, and a percutaneous pedicular screw fixation system^14^ as shown in Figure 1.

The mean operative times was 355 (275-420) minutes, and patients were discharged from the hospital after 3 (2-4) days. Mean blood loss was 215.8 (SD, 76.5) mL and none of the patients required blood transfusion. Only 1 among the 19 evaluated patients presented low-back pain after surgery and 11 had leg pain. Their work activities mainly involved manual labor, and our sample included 9 loaders, 4 machine operators, 3 office workers, 1 security guard, 1 waiter, and 1 mining technician. Only one of the office workers was employed in the public sector. Most patients (68.4%) did not return to work after surgery, and the median time for those that returned was 25 (2-67) months. The patient with the fastest return to work (2 months) was a female office worker, and among those who did not return to work (13 patients), 6 were loaders (46%). All patients received social security benefits after surgery.

Information related to the surgical procedure and the epidemiological data on the patients are presented on Table 1.

**Table 1:** Characteristics of the surgical procedure and clinical state of patients before and after surgery (n = 19).

Characteristics	n (%)	Mean ± SD or median (Q25-Q75)
Operated level		
1	5 (26.3)	-
2	14 (73.7)	-
Operated side		
Right	10 (52.6)	-
Left	9 (47.4)	-
Operative time (minutes)	-	355.0 (275.0-420.0)
Hemoglobin concentration before surgery (g/dL)	-	15.2+1.1
Hematocrit before surgery (%)		45.2+2.6
Hemoglobin concentration after surgery (g/dL)		11.4+1.8
Hematocrit after surgery (%)		34.5+4.0
Total bleeding (mL)		215.8+6.5
Length of stay (days)	-	3.0 (2.0-4.0)
Pain perception after surgery (a.u.)	-	1.0 (0.0-3.0)
Pain location after surgery		
No pain	7 (36.8)	-
Low-back	1 (5.3)	-
Leg	11 (57.9)	-
Work resumption		
No	13 (68.4)	-
Yes	6 (31.6)	-

a.u.: arbitrary units; SD: standard deviation.

**Figure 2:**
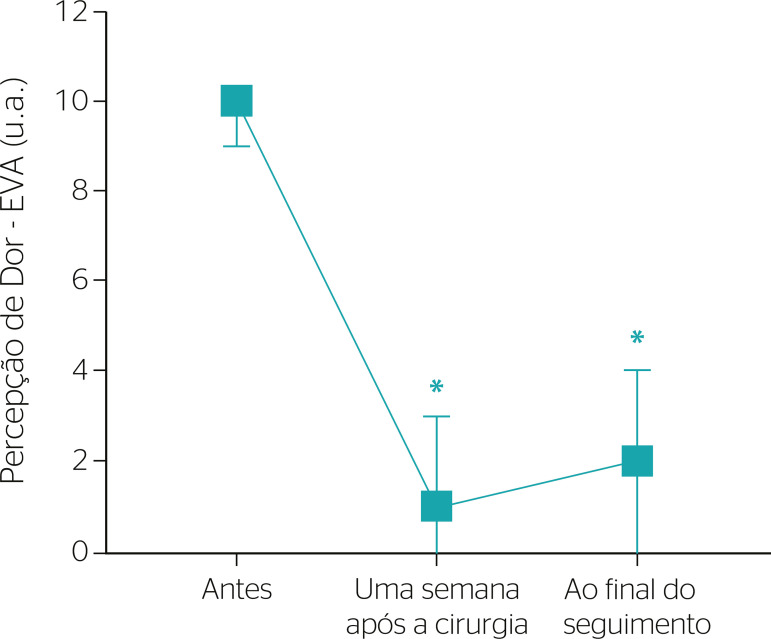
Pain perception before surgery, 1 week after surgery, and at the end of the follow-up period (n = 19). Data are presented as median and quartiles (Q25-Q75). * Statistically different from before surgery. VAS: visual analog scale; a.u.: arbitrary units.

The median value of pain perception before surgery was 10 (9-10) arbitrary units (a.u.) according to the VAS, and was reduced to 1 (0-3) a.u. after surgery. No statistical difference was noticed between the pain intensity perceived 1 week after the procedure (1 [0-3] a.u.) and at the end of the follow-up period (2 [0-4] a.u., χ^2^ [2] = 29.288, p < 0.001) (Figure 2).

**Figure 3:**
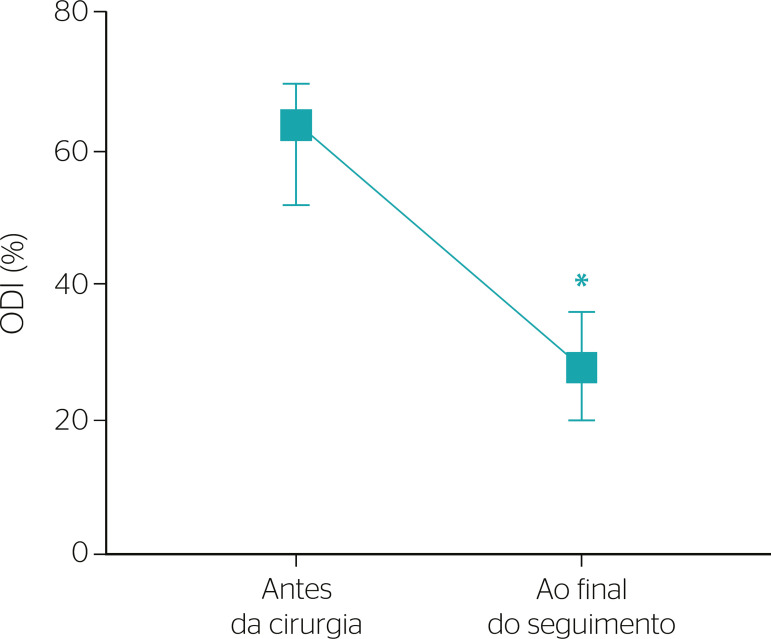
Oswestry disability indices (ODIs) before surgery and at the end of follow-up. Data are presented as median and quartiles (Q25-Q75). * Statistically different from before surgery.

When comparing functional status according to the ODI before and after follow-up, the scores showed a significant decrease (64 [52-70] vs. 28 [20 a 36]%; z = -3.503, p < 0.001] (Figure 3).

The evaluation of quality of life measured by the SF-36 indicated significant improvement regarding scores for physical functioning (z = -3.509; p < 0.001), role limitation due to physical problems (z = 3.535; p < 0.001), body pain (z = 3.624; p < 0.001), general health perception (z = 3.333; p = 0.001), and vitality (z = 3.247; p = 0.001). The social functioning (z = -0.700; p = 0.484) and mental health (z = -1.479; p = 0.139) scales were similar before surgery and at the end of our follow-up (Table 2).

**Table 2:** Quality of life as measured by the Medical Outcomes Short-Form Health Survey (SF-36) before surgery and at the end of follow-up (n = 19).

SF-36 scales	Before surgery	End of follow-up	p-value*
	n (%)	Median (Q25-Q75)	
Physical functioning	10 (0-25)	90 (50-100)	**< 0.001**
Role limitation due to physical problems	0 (0-0)	100 (25-100)	**< 0.001**
Body pain	0 (12-22)	62 (51-100)	**< 0.001**
General health perception	25 (5-35)	82 (57-100)	**0.001**
Vitality	25 (10-40)	80 (50-100)	**0.001**

* Numerical values in bold correspond to statistically significant differences. SD: standard deviation.

We had four patients that presented complications and required reintervention: a case of prosthesis migration, a case of subsidence leading to prosthesis removal, one infection, and one mispositioned screw. None of the cases required open surgery, and all complications were corrected with minimally invasive spine surgery; only 1 out of the 19 patients presented pseudarthrosis.

## DISCUSSION

Surgical procedures for low-back pain have been extensively studied and provide satisfactory long-term results. However, immediate postoperative periods still present drawbacks such as the pain and limitations on physical functioning experienced after open laminectomies on patients with spinal instability and peridural fibrosis.^15^

Our mean follow-up period was 47 (70-29) months. The reference work performed by Lee et al.^16^ reported a mean follow-up of 46 (12-123) months.

The mean period of clinical progression before surgery was 40.3 (7-132) months. This long period differs from the recommended 6 weeks^8^ due to delays in the patients’ access to specialized care. Early diagnosis is of crucial importance in osteoarticular injury because workers that receive this care have 13% higher chances of returning to work in comparison to those that have a delayed access to specialized care.^17^

Our mean operative time was 355 (275-420) minutes. Other studies reported a mean operative time of 113.5 (SD, 6.3) minutes (range, 105-120),^19^ illustrating that the learning curve for this procedure can be extensive. Nevertheless, the clinical recovery and early discharge (mean 3[2-4] days) of our patients was similar to that reported by Wang & Grossman,^20^ who reported patient discharge after 1.4 (SD, 1.3) overnight stays; therefore, our longer operative times did not affect early patient discharge. In addition, considering that our patients were discharged early after a mean 3 (2-4) days, we confirmed the data available on the literature regarding minimally invasive spine surgery: it presents several advantages over the traditional procedure, with less soft tissue damage and postoperative pain and a faster recovery. Altogether, these advantages provide economic advantages owing to shorter hospitalization times and a faster return to work.^3^

Our results indicate a mean blood loss of 215.8 (SD, 76.5) mL, which is similar to that reported by other percutaneous procedures: 238 mL (140-350).^19^ None of the patients required blood transfusions, which excluded the risks associated with this procedure. The complications encountered in our study are similar to those reported by the literature^20^: four cases required reintervention due to prosthesis migration, subsidence, infection, and a mispositioned screw. Nevertheless, none of them had to be subjected to conventional lumbar arthrodesis and all cases were corrected by minimally invasive spine surgery. Our results indicate that, despite requiring a high demand, as reported by other researchers,^21^ minimally invasive spine surgery achieves complete spinal fusion, representing a safe and effective alternative procedure.

When evaluating the return to work, clinical and functional parameters (expressed by VAS, ODI, and SF-36 scores) should be analyzed. We observed a significant improvement in postoperative recovery when comparing pain perception before surgery, 1 week after the procedure, and at the end of follow-up. Immediate and long-term pain were reduced since scores remained low after 47 months of follow-up even though the patients received social security benefits (Figure 2).

Similarly, some Brazilian studies reported that patients that received these benefits presented improved postoperative clinical and functional parameters when compared to those that did not receive social security benefits, even though their recovery period was longer.^3^ On the other hand, studies that used the same evaluation scores showed that patients that received social security benefits had worse clinical outcomes after lumbar surgery.^22^

The fact that there is no correlation between clinical improvement after surgery (according to the functional parameters measured by VAS, ODI, and SF-36) and the patients’ return to work has also been noticed by international studies, and could be related to a significant work incapacity already in the preoperative period and to low levels of education.^23^ Most of our patients (68.4%) did not return to work after surgery, and among those who did, the mean time for the return to work was 25 (2-67) months, in spite of satisfactory clinical results. All our patients received social security benefits after surgery.

Our findings (68.4% of patients did not return to work) are similar to those reported by Brazilian literature regarding workers that receive social security benefits (64%); an association between social security benefits and worse clinical outcomes is frequently reported when comparing patients who have these benefits with those who do not.^3^ Other national studies presented similar results and indicated that patients who received social security benefits had twice the chances of unsatisfactory surgery results when compared to those who did not have these benefits (43% vs 17%); this association remained consistent when results were grouped according to country or procedure. The authors concluded that the possibility of receiving social security benefits should be considered when performing studies on spine surgery procedures.^23^

Moreover, systematic reviews have shown that although 91% of the patients were discharged from the hospital 24 hours after surgery and the longest stay was of 3 days, 57% of those who had social security benefits only returned to work 48 months after surgery.^24^ In our study, the patient that presented the fastest return to work (2 months) was an office worker, and among those who did not return to work (13 patients), 6 were loaders (46%) and only 1 was not a manual laborer.

Several studies have shown that musculoskeletal disorders are the main cause of long-term sickness absence; the most frequently encountered problem is low-back pain caused by cumulative trauma, and the source of this symptom could include inadequate postures during work activities, high physical demand of manual labor, and lack of rest pauses.^25^ An association between manual labor professions and non-return to work after spine surgery is frequently reported, and available studies show even lower return-to-work rates (only 20% among manual labor workers) than those encountered in our work.^26^

These results indicate that the return to work after lumbar surgery is a global challenge. International studies that used the same measuring scales and provided work adaptations after surgery, such as ergonomic improvements, showed that good results regarding pain and postoperative function did not necessarily result in a high return-to-work rate, which was similar to our results. The authors thus concluded that a positive clinical outcome does not lead to a positive tendency of return to work after lumbar surgery.^27^ International studies have also shown similar results: excellent clinical results did not correlate with the return to work, and researchers could not evaluate work resumption because most patients remained in sickness absence for social reasons.^28^

These studies also corroborate these findings (up to 75% of patients subjected to lumbar spinal fusion did not return to work) and highlight the importance of evaluating social and work-related issues preoperatively. Authors have linked these results to the low educational levels of workers, the manual nature of their labor, and most importantly, to low salaries, since the quality of life of the workers who returned to work did not significantly change in comparison to those who did not return.^29^

## CONCLUSIONS

This study provides evidence that minimally invasive spine surgery achieved its objective (spinal fusion), representing a safe and effective option for different groups of workers even after 47 months of follow up; however, these results did not translate into a satisfactory return-to-work rate. Other factors such as low education levels, manual labor, low salaries, and the validity of social security benefits should be considered when planning surgical interventions for these patients.
